# The Effect of Caffeine on the Risk and Progression of Parkinson’s Disease: A Meta-Analysis

**DOI:** 10.3390/nu12061860

**Published:** 2020-06-22

**Authors:** Chien Tai Hong, Lung Chan, Chyi-Huey Bai

**Affiliations:** 1 Department of Neurology, Shuang-Ho Hospital, Taipei Medical University, New Taipei 23561, Taiwan; ct.hong@tmu.edu.tw; 2 Department of Neurology, School of Medicine, College of Medicine, Taipei Medical University, Taipei 11031, Taiwan; 3 School of Public Health, College of Public Health, Taipei Medical University, Taipei 11031, Taiwan; 4 Department of Public Health, School of Medicine, College of Medicine, Taipei Medical University, Taipei 11031, Taiwan; 5 Nutrition Research Center, Taipei Medical University Hospital, Taipei 11031, Taiwan

**Keywords:** caffeine, Parkinson’s disease, risk, progression, meta-analysis

## Abstract

Coffee and caffeine are speculated to be associated with the reduced risk of Parkinson’s disease (PD). The present study aimed to investigate the disease-modifying potential of caffeine on PD, either for healthy people or patients, through a meta-analysis. The electronic databases were searched using terms related to PD and coffee and caffeinated food products. Articles were included only upon fulfillment of clear diagnostic criteria for PD and details regarding their caffeine content. Reference lists of relevant articles were reviewed to identify eligible studies not shortlisted using these terms. In total, the present study enrolled 13 studies, nine were categorized into a healthy cohort and the rest into a PD cohort. The individuals in the healthy cohort with regular caffeine consumption had a significantly lower risk of PD during follow-up evaluation (hazard ratio (HR) = 0.797, 95% CI = 0.748–0.849, *p* < 0.001). The outcomes of disease progression in PD cohorts included dyskinesia, motor fluctuation, symptom onset, and levodopa initiation. Individuals consuming caffeine presented a significantly lower rate of PD progression (HR = 0.834, 95% CI = 0.707–0.984, *p* = 0.03). In conclusion, caffeine modified disease risk and progression in PD, among both healthy individuals or those with PD. Potential biological benefits, such as those obtained from adenosine 2A receptor antagonism, may require further investigation for designing new drugs.

## 1. Introduction

Parkinson’s disease (PD) is one of the most common neurodegenerative diseases, second only to Alzheimer’s disease (AD). Risk factors for PD include genetic mutations, environmental toxins, and lifestyle [1]. An epidemiological study reported some protective factors for PD worldwide, such as female sex, physical activity, and smoking [2]. The consumption of coffee or caffeinated food is associated with the reduction of the risk of PD. Patients with PD are less frequent habitual consumers of caffeinated food [3,4]. The consumption of either tea or coffee exhibited similar effects on the reduction of the risk of PD [5]. In a similar manner, the protective effect of coffee was also noted in dementia and AD [6], whereby caffeine reversed the cognitive impairment and decreased the amyloid burden in transgenic AD mice model [7].

Caffeine is an adenosine A2A receptor antagonist [8]. Different types of adenosine receptors (A1, A2A, A2B, and A3) are widely distributed in the brain. Adenosine A2A receptors are coupled with G-proteins and exclusively expressed in dopaminergic neurons. The activation of adenosine A2A receptors causes an increase in intracellular cAMP levels and the extracellular release of glutamate, resulting in neural excitotoxicity [9]. The neuroprotective effects of caffeine involved the antagonism of the adenosine A2A receptor, down-regulating the down streaming phosphatidylinositol 3-kinase (PI3K)/protein kinase B (AKT) signaling pathway, and avoiding excessive calcium releasing-related neurotoxicity and neuroinflammation [10], which has been experimentally demonstrated in several in vivo models of PD [11,12,13,14].

Whether caffeine can reduce the risk and halt the progression of PD remains unclear. In large-scale cohort studies, caffeine consumption was inconsistently associated with a low risk of PD during follow-up [15,16,17,18]. However, among patients diagnosed with PD, the administration of caffeine tablets did not modify the disease course [19]. Furthermore, caffeine metabolism varies among patients with PD [20], thus potentially resulting in inconsistent protective effects. This study investigated the association between caffeine and PD progression. Considering disease progression was the primary temporal outcome, only cohort studies rather than case–control studies were included herein, because case–control studies cannot delineate this temporal association. 

## 2. Methods 

### 2.1. Literature Search Strategy

All relevant articles published in English between 1 January 1990, and 31 December 2019 were identified by searching PubMed, BioMed Central, Medline, and Google Scholar. Details regarding search terms are provided in supplementary data. Moreover, the reference lists of relevant articles were reviewed to identify eligible studies not derived using these search terms. 

### 2.2. Inclusion and Exclusion of the Literature

**Figure 1:**
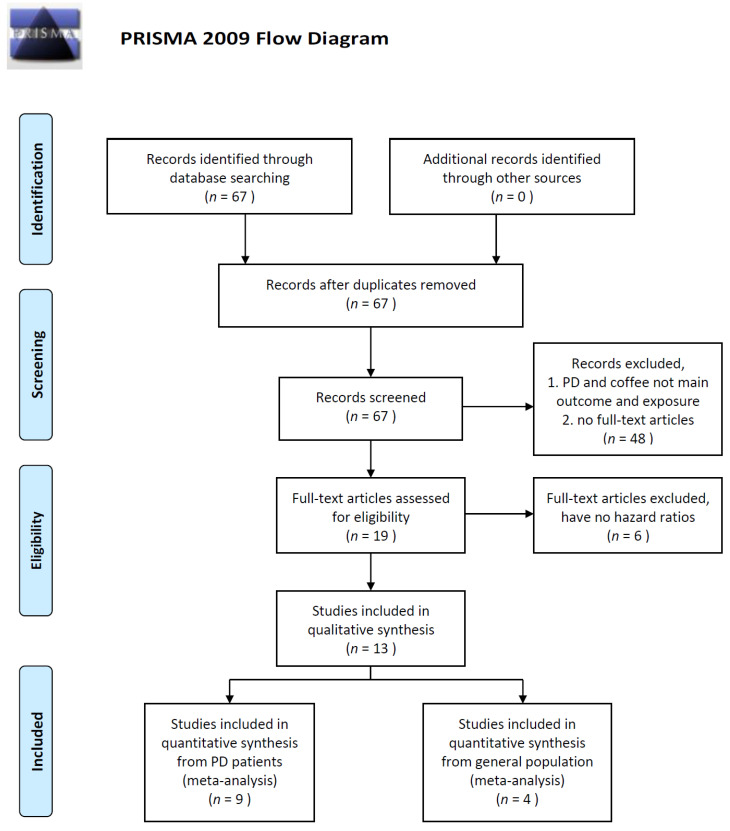
A schematic representation of the literature search.

Inclusion criteria were as follows: (1) clear definition of PD diagnosis; (2) clear definition regarding the quantity of caffeine, coffee, or tea consumption; (3) cohort study published as an original article, case series, or letter to the editor; (4) sample size of ≥50 individuals; and (5) published in English. After excluding nonqualified studies, 19 studies were entered the full-article assessment process and another 6 studies were excluded due to the lack of hazard ratio. Finally, 13 studies were included into qualitative synthesis. We further segregated the remaining 13 studies into two categories: the healthy cohort including studies (*n* = 9) that recruited individuals without previous diagnosis of PD, wherein PD diagnosis was performed during follow-up evaluation, and the PD cohort including studies (*n* = 4) on individuals with PD already presenting motor symptoms, wherein PD progression was monitored. The selection process is illustrated in Figure 1.

### 2.3. Data Extraction

The following data were extracted: name of the first author; year of publication; country and location; study design; the original cohort or clinical trial; the starting time of cohort; diagnostic criteria for PD; the assessment of caffeine consumption; the amount of coffee or caffeine consumption; mean follow-up period of time; the outcome assessment time; and the outcome of the PD progression. All data were independently reviewed by three investigators (BAI CH, Hong CT, and Chan L), and conflicts were resolved through a consensus. Assessing of quality of all studies were done by three investigators (BAI CH, Hong CT, and Chan L) based on the Newcastle–Ottawa Scale. The study was recommended (>7) by at least 2 investigators into this study as candidate. Data from these 13 candidate studies were independently extracted by two investigators (BAI CH and FAN YC). 

### 2.4. Statistical Analysis

The hazard ratio (HR) was determined, and 95% CIs were calculated on the basis of a binomial assumption. I^2^ was used to assess heterogeneity across studies. All statistical analyses were performed using SAS software (version 9.3; Statistical Analysis System, SAS.com, USA). All reported probability (*p*) values were two-sided, with *p* < 0.05 considered statistically significant. 

### 2.5. Data Availability

The present study was a meta-analysis and all the studies enrolled into analysis can be found through the provided searching strategy. 

## 3. Results

Among the nine studies included in the healthy cohort (Table 1) [15,16,17,18,21,22,23,24,25], five were conducted in the United States, three in Scandinavia, and one in Singapore. Some studies were large-scale, long-term, population-based epidemiological cohort studies, and the others were specific for individuals with certain characteristics (nurses, healthcare professions, twins, and ancestry of migrants). Caffeine consumption was evaluated using questionnaires, either detailed and comprehensive or simple ones. Five of them investigated overall dietary habits, including coffee, tea, cola, and chocolate consumption, by using the transforming formula. The rest of them only recorded the daily consumption of coffee or tea. PD was diagnosed through either self-report and confirmation of medical records or from the national health care database. Two studies separately reported the results for men and women, and another study reported data only for women. 

**Figure 2:**
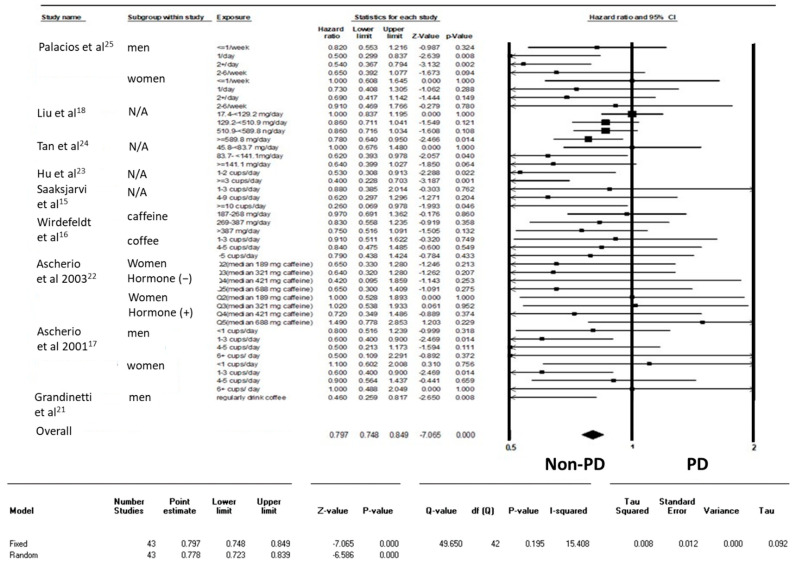
Forest plot illustrating the hazard ratio (HR) of Parkinson’s disease (PD) among healthy individuals from cohort studies.

Most of the included studies categorized caffeine consumption as degree 4–5 based on the amount of caffeine or the number of cups of coffee per day. Only one study simply provided options of “yes” and “no” with regard to regular coffee consumption. Considering the difficulty in transforming the actual caffeine consumption among studies, this study considered results of all individuals consuming coffee at all degrees and considered the no-exposure group as a reference group to determine the HR. Overall, 43 results extracted from nine studies were analyzed herein. Caffeine consumption was significantly associated with a lower risk of developing remarkable symptoms for the diagnosis of PD during the follow-up period of time (HR = 0.797, 95% CI: 0.748–0.849, *p* < 0.001; Figure 2).

**Figure 3:**
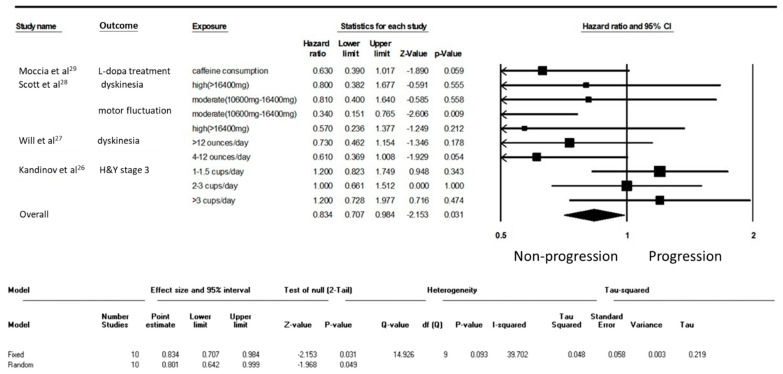
Forest plot illustrating the hazard ratio (HR) of progression of Parkinson’s disease (PD) among individuals with early-stage PD.

This study analyzed the effect of caffeine on patients with PD (Table 2) [26,27,28,29]. Among the four studies in the PD cohort, three were conducted in European countries and one in the United States. Patients with PD were in the early stage of the disease. Similar to the healthy cohort, levels of caffeine consumption were assessed through either comprehensive questionnaires or simple questions. The four studies set different parameters for PD progression, including the initiation of levodopa, levodopa-induced motor complications, and the transition to Hoehn and Yahr stage III. The average follow-up duration ranged 4 to 10.3 years. Finally, 10 results were extracted from these 4 studies. Caffeine composition among patients at an early stage of PD significantly decelerated PD progression (HR = 0.834, 95% CI = 0.707–0.984, *p* = 0.03; Figure 3).

## 4. Discussion

The results of this study showed that among both healthy individuals and patients with PD, caffeine consumption was significantly associated with a lower HR for the risk or progression of PD, respectively. Considering that steady neurodegeneration in PD precedes the onset of motor symptoms for decades and persists thereafter [30], caffeine was speculated to have disease-modifying potential throughout the course of the disease in this study. Compared with data obtained from case–control studies, data obtained from a combination of multiple cohorts were more likely to demonstrate the beneficial causal relationship between caffeine consumption and the risk of PD.

The potential neuroprotective effect of caffeine consumption against PD was noted on the basis of case–control epidemiological studies. Although another component in coffee, that is, eicosanoyl-5-hydroxytryptamide, is believed to protect against neurodegeneration [31], a similar association found for tea consumption further corroborated the finding that caffeine is the key protective agent in coffee [32]. Instead of psychostimulation, caffeine antagonizes the adenosine A2A receptor. In the central nervous system, the adenosine A2A receptor is exclusively expressed in dopaminergic neurons, and the activation of the adenosine A2A receptor triggers the cAMP-protein kinase A-dependent elevation of intracellular calcium and release of glutamate [33]. Excessive intracellular calcium and glutamate levels are responsible for excitotoxicity in neurodegenerative diseases, including PD [34]. The adenosine A2A receptor is also involved in neuroinflammation-mediated neuronal dysfunction and degeneration [35]. Istradefylline, an FDA-approved adenosine A2A receptor antagonist currently used for treating PD, reduces off time and improves the motor symptoms of patients with PD albeit with complications including the exacerbation of dyskinesia [36]. The neuroprotective effect of istradefylline has been further described in in vivo studies [37,38,39] but not in clinical.

However, large-scale cohort studies focused on the healthy population, did not consistently demonstrate the risk-reduction effect of caffeine on PD. Studies investigating the effect of caffeine on PD progression among PD patients were also not fruitful. The quantification of daily caffeine consumption is most challenging for studies intending to investigate disease-modifying effects. Except caffeine tablets, the assessment of the daily intake of caffeinated beverages and food products requires a formula for transformation. A structured dietary interview is usually necessary to obtain semi-quantitative data regarding daily caffeine intake. Considering that adults usually adhere to their dietary preferences, the interview would yield reliable data regarding long-term levels of caffeine consumption. However, coffee, tea, cola, and chocolate in different styles, brands, or countries (even areas) have different caffeine contents. Moreover, genetic polymorphism, sex, and heterogeneity in caffeine metabolism also influence the effects of caffeine [20,40,41]. Herein, most studies segregated their participants on the basis of caffeine consumption by relative gradients, thus introducing a slight variation among the enrolled studies. This relative but not absolute grouping deters inter-study comparisons and the obtainment of consistent findings.

Defining PD progression is challenging and problematic. Among healthy individuals, tremors may be visible and can be recognized early. However, the remaining cardinal motor symptoms, including rigidity, bradykinesia, and postural instability, are ambiguous. Most individuals with PD were either misdiagnosed or underwent unnecessary treatment years before reaching a final diagnosis [42]. This delay deters the assessment of disease progression for the cohort study recruiting healthy participants. Regarding disease progression among patients with PD, off-status motor function is the major parameter among clinical trials. However, responses to one-night washout are variable, and the effect of levodopa may last for 2 weeks [43]. Moreover, if the intervention itself causes certain symptomatic effects in conjunction, similar to rasagiline or caffeine, it would be challenging to distinguish between disease modification and symptomatic effect [44]. However, the onset of motor fluctuation and dyskinesia have been considered markers of disease progression among patients with PD. Nevertheless, the degeneration of dopaminergic neurons is not the only underlying factor [45]. The dosage of levodopa, the prescription of dopamine agonists, or amantadine in the early stage of the disease also influences the onset of motor complications [46,47,48]. One study included herein considered the initiation of levodopa treatment as a marker for disease progression, which was highly influenced by the subjective and objective conditions of patients with PD [49]. Young or old; employed, self-employed, or retired individuals and the self-expectation had an influence on levodopa initiation. These aforementioned issues deter the accurate assessment of disease progression, thus yielding inconsistent disease-modifying effects of caffeine or any other interventions.

The strength of this study is the delineation of the disease-modifying effect of caffeine on PD. The inclusion of exclusive cohort studies was superior to case–control studies owing to the potential temporal association between caffeine and PD, and the prevention of recall bias on the dietary habit. Furthermore, this study pooled all the HR of PD from moderate-to-high levels of caffeine consumption together and determined the lower limit as reference, thus eliminating the ambiguous cut-off level of daily caffeine intake in numerous studies. This blurred “beneficial dosage of caffeine” varied among studies and confounded clinicians and the population. Moreover, no remarkable J-shaped curve was previously obtained for the risk of PD and caffeine consumption, thus yielding an upper limit of permissible caffeine consumption. The present results indicate that caffeine consumption potentially alters the PD risk and progression among both healthy individuals and those with PD, and this concept is easier to pick up by general population and health professions.

This study has some limitations. First, considering the diagnosis of PD heavily relying on the development of the motor symptoms, which may be delayed for decades after the beginning of the neurodegeneration, the utilization of the diagnosis of PD as outcome assessment in the healthy cohorts may bias by either under or overvaluation. Second, variations in the levels of caffeine consumption among studies undoubtedly introduced heterogeneity among studies. Certain studies focused on caffeinated food products, and another study focused only on coffee or tea. The effect of some promising ingredients in coffee, such as eicosanoyl-5-hydroxytryptamide [31] or methylxanthine [50] had not been investigated in the present study due to the lack of standard assessment, such as the dietary questionnaires for the amount of daily intake. None of the studies included in this research focused on the effect of pure caffeine tablets, which would directly demonstrate the effects of caffeine rather than the mixed effects of caffeinated food products. Based on this information provided from the dietary questionnaires, it was not possible to define the optimal daily dosage of caffeine and the food source of caffeine. Third, instead of coffee or caffeine, some factors are known to affect the risk of PD, such as diabetes, pesticide exposure and the well-water drinking [51]. Female is well-known for the lower risk of PD, and the protective role of sex hormone is speculated [52]. However, in the present study, two cohorts separated the results between men and women, which revealed no significant heterogenicity. Meanwhile, one study also sub-grouped female participants based on the hormone replacement therapy, and there was no remarkable heterogenicity either. Moreover, several genetic and environmental factors interact with caffeine, and life style, socioeconomic status, exercise, high-fat diet and alcohol consumption may also be associated with the habitual coffee drinking. There was no clear information about those environmental factors from the included studies for the authors to adjust those possible confounding factors. Lastly, genetic polymorphism may affect the metabolism of caffeine, but to the best of our knowledge, only case-control studies were found to investigate the gene–caffeine interaction in PD [53,54,55], which did not fit into the inclusion criteria of the present meta-analysis.

In conclusion, this meta-analysis shows that caffeine is associated with a low risk of developing PD in healthy individuals and the deceleration in the progression of motor symptoms in patients with PD. Additional studies are required to investigate not only the optimal daily dosage and food source of caffeine for PD, but also the possible mechanisms underlying the bioprotective effects of caffeine on PD. Among individuals with PD, caffeine intake should be encouraged if adverse effects are tolerable.

## Figures and Tables

**Table 1:** List of the included cohort study.

Study Name	Country	Original Cohort (Established-Last Outcome Assessment)	*n*	Assessment Caffeine Consumption	Amount of Caffeine Consumption	The Diagnosis of PD
Ascherio et al. [17]	USA	Health Professionals’ Follow-Up Study and Nurses’ Health Study (1976 and 1986/1994)	135,916	Semiquantitative food-frequency questionnaire (SFFQ)	Caffeine was 137 mg per cup of coffee, 47 mg per cup of tea, 46 mg per can or bottle of cola beverage, and 7 mg per serving of chocolate candy.	Self-report and medical records
Ascherio et al. [22]	USA	Nurses’ Health Study (1976/1998)	121,700 women	Semiquantitative food-frequency questionnaire (SFFQ)	Caffeine was 137 mg per cup of coffee, 47 mg per cup of tea, 46 mg per can or bottle of cola beverage, and 7 mg per serving of chocolate candy.	Medical records
Grandinetti et al. [21]	USA	Honolulu Heart Program-Japanese and Okinawan ancestry (1965/1991)	8006 men	Questionnaires	NA	Medical records
Hu et al. [23]	FIN	Four independent cross-sectional population surveys were carried out in five geographic areas of Finland in 1982, 1987, 1992, and 1997 (1982/2002)	29,335	Self-administered questionnaire	Cups of coffee	National Social Insurance Institution’s Register
Liu et al. [18]	USA	NIH-AARP Diet and Health Study (1995/2010)	566,401	Diet History Questionnaire	Nutrient calculation: 1994–1996 US Department of Agriculture’s Continuing Survey of Food Intakes by Individuals.	Interview and copy of medical records
Palacios et al. [25]	USA	CPS II–Nutrition cohort (1992/2007)	184,190	Food Frequency Questionnaire	137 and 47 mg per cup of coffee and tea, respectively, 46 mg per can or bottle of cola; and 7 mg per serving of chocolate.	Interview and copy of medical records
Sääksjärvi et al. [15]	FIN	Finnish Mobile Clinic Health Examination Survey (1973/1994)	7246	Self-administered, health questionnaire	Cups of coffee	National Social Insurance Institution’s Register
Tan et al. [24]	SG	Singapore Chinese Health Study (1993/2005)	63,257	A validated, semiquantitative food frequency section questionnaire	Singapore Food Composition Table, a food-nutrient database that lists the levels of 96 Nutritive/nonnutritive components (including caffeine) per 100 g of cooked food and beverages	Interview and linkage database to medical record
Wirdefeldt et al. [16]	SE	Swedish Twin Registry (1961 and 1973/without clear mentioning)	52,149	Questionnaires	Did not provide the formula	Inpatient Discharge Register and Cause of Death Register

**Table 2:** List of the included studies on the progression of Parkinson’s disease (PD).

Study Name	Country	Number of PD	Stage of PD	Assessment Caffeine Consumption	Amount of Caffeine Consumption	Mean Follow-Up Period of Time	Outcome as the Progression of PD
Kandinov et al. [26]	IL	278	Onset of PD motor symptoms	Interview	The number of cups of coffee per day	10.3 years	Time from onset to Hoehn and Yahr stage 3
Moccia et al. [29]	IL	79	de novo, drug naïve	Caffeine Consumption Questionnaire	i.e., Espresso 1oz = 50 mg caffeine	4 years	Starting L-dopa treatment
Scott et al. [28]	GB	183	Newly diagnosed	Verbal interview about the average level of exposure before baseline	Cups of tea: 47 mg caffeine Cup of coffee: 62 mg caffeine	59 months	1.Motor fluctuation 2.Dyskinesia
Wills et al. [27]	US	228	Early PD	questionnaire assessing both current (“in the past week”) and prior (“on average over the past 5 years”) caffeine intake	Coffee (85 mg caffeine/5 oz) Tea (36 mg caffeine/5 oz) Soda (45 mg caffeine/12 oz)	5.5 years	Dyskinesia

## Supplementary Materials

The following are available online at https://www.mdpi.com/2072-6643/12/6/1860/s1.

## Abbreviations

PD	Parkinson’s disease
HR	hazard ratio
CI	confidence interval

## References

[1] Wirdefeldt K., Adami H.-O., Cole P., Trichopoulos D., Mandel J. (2011). Epidemiology and etiology of Parkinson’s disease: A review of the evidence. Eur. J. Epidemiol..

[2] Kieburtz K., Wunderle K.B. (2013). Parkinson’s disease: Evidence for environmental risk factors. Mov. Disord..

[3] Jiméanez-Jiméanez F.J., Mateo D., Giméanez-Roldan S. (1992). Premorbid smoking, alcohol consumption, and coffee drinking habits in Parkinson’s disease: A case-control study. Mov. Disord..

[4] Hellenbrand W., Boeing H., Robra B.-P., Seidler A., Vieregge P., Nischan P., Joerg J., Oertel W.H., Schneider E., Ulm G. (1996). Diet and Parkinson’s disease II, A possible role for the past intake of specific nutrients: Results from a self-administered food-frequency questionnaire in a case-control study. Neurology.

[5] Tan E.K., Tan C., Fook-Chong S.M., Lum S.Y., Chai A., Chung H., Shen H., Zhao Y., Teoh M.L., Yih Y. (2003). Dose-dependent protective effect of coffee, tea, and smoking in Parkinson’s disease: A study in ethnic Chinese. J. Neurol. Sci..

[6] Eskelinen M.H., Kivipelto M. (2010). Caffeine as a protective factor in dementia and Alzheimer’s disease. J. Alzheimers Dis..

[7] Arendash G.W., Mori T., Cao C., Mamcarz M., Runfeldt M., Dickson A., Rezai-Zadeh K., Tane J., Citron B.A., Lin X. (2009). Caffeine reverses cognitive impairment and decreases brain amyloid-beta levels in aged Alzheimer’s disease mice. J. Alzheimers Dis..

[8] Fredholm B.B., Battig K., Holmen J., Nehlig A., Zvartau E.E. (1999). Actions of caffeine in the brain with special reference to factors that contribute to its widespread use. Pharmacol. Rev..

[9] Carpenter B., Lebon G. (2017). Human Adenosine A(2A) Receptor: Molecular Mechanism of Ligand Binding and Activation. Front. Pharmacol..

[10] Kolahdouzan M., Hamadeh M.J. (2017). The neuroprotective effects of caffeine in neurodegenerative diseases. CNS Neurosci. Ther..

[11] Chen J.F., Xu K., Petzer J.P., Staal R., Xu Y.H., Beilstein M., Sonsalla P.K., Castagnoli K., Castagnoli N., Schwarzschild M.A. (2001). Neuroprotection by caffeine and A(2A) adenosine receptor inactivation in a model of Parkinson’s disease. J. Neurosci..

[12] Bove J., Serrats J., Mengod G., Cortes R., Tolosa E., Marin C. (2005). Neuroprotection induced by the adenosine A2A antagonist CSC in the 6-OHDA rat model of parkinsonism: Effect on the activity of striatal output pathways. Exp. Brain Res..

[13] Kelsey J.E., Langelier N.A., Oriel B.S., Reedy C. (2009). The effects of systemic, intrastriatal, and intrapallidal injections of caffeine and systemic injections of A2A and A1 antagonists on forepaw stepping in the unilateral 6-OHDA-lesioned rat. Psychopharmacology.

[14] Reyhani-Rad S., Mahmoudi J. (2016). Effect of adenosine A2A receptor antagonists on motor disorders induced by 6-hydroxydopamine in rat. Acta Cir. Bras..

[15] Saaksjarvi K., Knekt P., Rissanen H., Laaksonen M.A., Reunanen A., Mannisto S. (2008). Prospective study of coffee consumption and risk of Parkinson’s disease. Eur. J. Clin. Nutr..

[16] Wirdefeldt K., Gatz M., Pawitan Y., Pedersen N.L. (2005). Risk and protective factors for Parkinson’s disease: A study in Swedish twins. Ann. Neurol..

[17] Ascherio A., Zhang S.M., Hernan M.A., Kawachi I., Colditz G.A., Speizer F.E., Willett W.C. (2001). Prospective study of caffeine consumption and risk of Parkinson’s disease in men and women. Ann. Neurol..

[18] Liu R., Guo X., Park Y., Huang X., Sinha R., Freedman N.D., Hollenbeck A.R., Blair A., Chen H. (2012). Caffeine intake, smoking, and risk of Parkinson disease in men and women. Am. J. Epidemiol..

[19] Postuma R.B., Lang A.E., Munhoz R.P., Charland K., Pelletier A., Moscovich M., Filla L., Zanatta D., Romenets S.R., Altman R. (2012). Caffeine for treatment of Parkinson disease: A randomized controlled trial. Neurology.

[20] Fujimaki M., Saiki S., Li Y., Kaga N., Taka H., Hatano T., Ishikawa K.-I., Oji Y., Mori A., Okuzumi A. (2018). Serum caffeine and metabolites are reliable biomarkers of early Parkinson disease. Neurology.

[21] Grandinetti A., Morens D.M., Reed D., MacEachern D. (1994). Prospective study of cigarette smoking and the risk of developing idiopathic Parkinson’s disease. Am. J. Epidemiol..

[22] Ascherio A., Chen H., Schwarzschild M.A., Zhang S.M., Colditz G.A., Speizer F.E. (2003). Caffeine, postmenopausal estrogen, and risk of Parkinson’s disease. Neurology.

[23] Hu G., Bidel S., Jousilahti P., Antikainen R., Tuomilehto J. (2007). Coffee and tea consumption and the risk of Parkinson’s disease. Mov. Disord..

[24] Tan L.C., Koh W.P., Yuan J.M., Wang R., Au W.L., Tan J.H., Tan E.K., Yu M.C. (2008). Differential effects of black versus green tea on risk of Parkinson’s disease in the Singapore Chinese Health Study. Am. J. Epidemiol..

[25] Palacios N., Gao X., McCullough M.L., Schwarzschild M.A., Shah R., Gapstur S., Ascherio A. (2012). Caffeine and risk of Parkinson’s disease in a large cohort of men and women. Mov. Disord..

[26] Kandinov B., Giladi N., Korczyn A.D. (2009). Smoking and tea consumption delay onset of Parkinson’s disease. Parkinsonism Relat. Disord..

[27] Wills A.-M.A., Eberly S., Tennis M., Lang A.E., Messing S., Togasaki D., Tanner C.M., Kamp C., Chen J.-F., Oakes D. (2013). Study, Caffeine consumption and risk of dyskinesia in CALM-PD. Mov. Disord. Off. J. Mov. Disord. Soc..

[28] Scott N.W., Macleod A.D., Counsell C.E. (2016). Motor complications in an incident Parkinson’s disease cohort. Eur. J. Neurol..

[29] Moccia M., Erro R., Picillo M., Vitale C., Longo K., Amboni M., Pellecchia M.T., Barone P. (2016). Caffeine consumption and the 4-year progression of de novo Parkinson’s disease. Parkinsonism Relat. Disord..

[30] Vingerhoets F.J., Snow B.J., Lee C.S., Schulzer M., Mak E., Calne D.B. (1994). Longitudinal fluorodopa positron emission tomographic studies of the evolution of idiopathic parkinsonism. Ann. Neurol..

[31] Yan R., Zhang J., Park H.J., Park E.S., Oh S., Zheng H., Junn E., Voronkov M., Stock J.B., Mouradian M.M. (2018). Synergistic neuroprotection by coffee components eicosanoyl-5-hydroxytryptamide and caffeine in models of Parkinson’s disease and DLB. Proc. Natl. Acad. Sci. USA.

[32] Li F.-J., Ji H.-F., Shen L. (2012). A meta-analysis of tea drinking and risk of Parkinson’s disease. Sci. World J..

[33] Wei C.J., Li W., Chen J.-F. (2011). Normal and abnormal functions of adenosine receptors in the central nervous system revealed by genetic knockout studies. Biochim. Biophys. Acta (BBA) Biomembr..

[34] Ambrosi G., Cerri S., Blandini F. (2014). A further update on the role of excitotoxicity in the pathogenesis of Parkinson’s disease. J. Neural. Transm..

[35] Machado-Filho J.A., Correia A.O., Montenegro A.B., Nobre M.E., Cerqueira G.S., Neves K.R., Mda G.N., Cavalheiro E.A., Brito G.A., Viana G.S. (2014). Caffeine neuroprotective effects on 6-OHDA-lesioned rats are mediated by several factors, including pro-inflammatory cytokines and histone deacetylase inhibitions. Behav. Brain Res..

[36] Rascol O., Perez-Lloret S., Ferreira J.J. (2015). New treatments for levodopa-induced motor complications. Mov. Disord..

[37] Golembiowska K., Wardas J., Noworyta-Sokolowska K., Kaminska K., Gorska A. (2013). Effects of adenosine receptor antagonists on the in vivo LPS-induced inflammation model of Parkinson’s disease. Neurotox. Res..

[38] Bibbiani F., Oh J.D., Petzer J.P., Castagnoli N., Chen J.F., Schwarzschild M.A., Chase T.N. (2003). A2A antagonist prevents dopamine agonist-induced motor complications in animal models of Parkinson’s disease. Exp. Neurol..

[39] Grondin R., Bedard P.J., Tahar A.H., Gregoire L., Mori A., Kase H. (1999). Antiparkinsonian effect of a new selective adenosine A2A receptor antagonist in MPTP-treated monkeys. Neurology.

[40] Arab L., Biggs M.L., O’Meara E.S., Longstreth W.T., Crane P.K., Fitzpatrick A.L. (2011). Gender differences in tea, coffee, and cognitive decline in the elderly: The Cardiovascular Health Study. J. Alzheimers Dis..

[41] Yang A., Palmer A.A., de Wit H. (2010). Genetics of caffeine consumption and responses to caffeine. Psychopharmacology.

[42] Breen D.P., Evans J.R., Farrell K., Brayne C., Barker R.A. (2013). Determinants of delayed diagnosis in Parkinson’s disease. J. Neurol..

[43] Fahn S., Oakes D., Shoulson I., Kieburtz K., Rudolph A., Lang A., Olanow C.W., Tanner C., Marek K. (2004). Levodopa and the progression of Parkinson’s disease. N. Engl. J. Med..

[44] Olanow C.W., Rascol O., Hauser R., Feigin P.D., Jankovic J., Lang A., Langston W., Melamed E., Poewe W., Stocchi F. (2009). A double-blind, delayed-start trial of rasagiline in Parkinson’s disease. N. Engl. J. Med..

[45] Kelly M.J., Lawton M.A., Baig F., Ruffmann C., Barber T.R., Lo C., Klein J.C., Ben-Shlomo Y., Hu M.T. (2019). Predictors of motor complications in early Parkinson’s disease: A prospective cohort study. Mov. Disord..

[46] Wu T.L., Wang C.C., Lin F.J., Wu R.M. (2018). The association between early treatment with amantadine and delayed onset of levodopa-induced dyskinesia in patients with Parkinson’s disease. Parkinsonism Relat. Disord..

[47] Group P.S. (2000). Pramipexole vs Levodopa as Initial Treatment for Parkinson DiseaseA Randomized Controlled Trial. JAMA.

[48] Olanow C.W., Kieburtz K., Rascol O., Poewe W., Schapira A.H., Emre M., Nissinen H., Leinonen M., Stocchi F. (2013). Factors predictive of the development of Levodopa-induced dyskinesia and wearing-off in Parkinson’s disease. Mov. Disord..

[49] Stocchi F., Vacca L., Radicati F.G. (2015). How to optimize the treatment of early stage Parkinson’s disease. Transl. Neurodegener..

[50] Oñatibia-Astibia A., Franco R., Martínez-Pinilla E. (2017). Health benefits of methylxanthines in neurodegenerative diseases. Mol. Nutr. Food Res..

[51] Martino R., Candundo H., Lieshout P.v., Shin S., Crispo J.A.G., Barakat-Haddad C. (2017). Onset and progression factors in Parkinson’s disease: A systematic review. NeuroToxicology.

[52] Pinares-Garcia P., Stratikopoulos M., Zagato A., Loke H., Lee J. (2018). Sex: A Significant Risk Factor for Neurodevelopmental and Neurodegenerative Disorders. Brain Sci..

[53] Simon D.K., Wu C., Tilley B.C., Lohmann K., Klein C., Payami H., Wills A.M., Aminoff M.J., Bainbridge J., Dewey R. (2017). Caffeine, creatine, GRIN2A and Parkinson’s disease progression. J. Neurol. Sci..

[54] Chuang Y.H., Lill C.M., Lee P.C., Hansen J., Lassen C.F., Bertram L., Greene N., Sinsheimer J.S., Ritz B. (2016). Gene-Environment Interaction in Parkinson’s Disease: Coffee, ADORA2A, and CYP1A2. Neuroepidemiology.

[55] Kim I.Y., O’Reilly É J., Hughes K.C., Gao X., Schwarzschild M.A., McCullough M.L., Hannan M.T., Betensky R.A., Ascherio A. (2018). Interaction between caffeine and polymorphisms of glutamate ionotropic receptor NMDA type subunit 2A (GRIN2A) and cytochrome P450 1A2 (CYP1A2) on Parkinson’s disease risk. Mov. Disord..

