# Correction: Hong et al. The Effect of Caffeine on the Risk and Progression of Parkinson’s Disease: A Meta-Analysis. *Nutrients* 2020, *12*, 1860

**DOI:** 10.3390/nu15030699

**Published:** 2023-01-30

**Authors:** Chien Tai Hong, Lung Chan, Chyi-Huey Bai

**Affiliations:** 1 Department of Neurology, Shuang-Ho Hospital, Taipei Medical University, New Taipei 23561, Taiwan; 2 Department of Neurology, School of Medicine, College of Medicine, Taipei Medical University, Taipei 11031, Taiwan; 3 School of Public Health, College of Public Health, Taipei Medical University, Taipei 11031, Taiwan; 4 Department of Public Health, School of Medicine, College of Medicine, Taipei Medical University, Taipei 11031, Taiwan; 5 Nutrition Research Center, Taipei Medical University Hospital, Taipei 11031, Taiwan

## Error in Figure/Table

In the original publication [1], there were some mistakes in Table 1 and Table 2 and Figure 2 and Figure 3 as published. The citation number in those aforementioned Tables and Figures was incorrect. The corrected Table 1 and Table 2 and Figure 2 and Figure 3 appear below. 

The authors apologize for any inconvenience caused and state that the scientific conclusions are unaffected. This correction was approved by the Academic Editor. The original publication has also been updated.

## Figures and Tables

**Figure 2:**
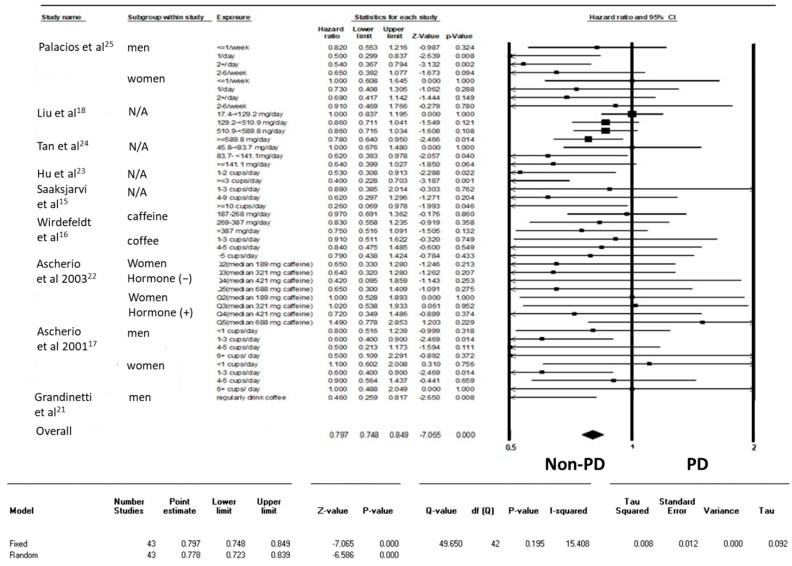
Forest plot illustrating the hazard ratio (HR) of Parkinson’s disease (PD) among healthy individuals from cohort studies.

**Figure 3:**
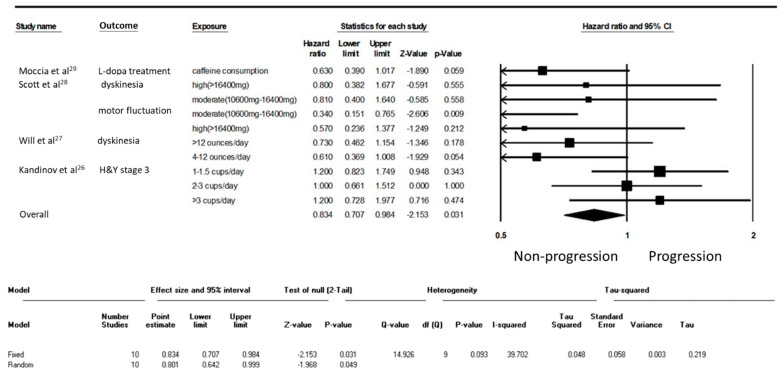
Forest plot illustrating the hazard ratio (HR) of progression of Parkinson’s disease (PD) among individuals with early-stage PD.

**Table 1:** List of the included cohort study.

Study Name	Country	Original Cohort (Established-Last Outcome Assessment)	*n*	Assessment Caffeine Consumption	Amount of Caffeine Consumption	The Diagnosis of PD
Ascherio et al. [17]	USA	Health Professionals’ Follow-Up Study and Nurses’ Health Study (1976 and 1986/1994)	135,916	Semiquantitative food-frequency questionnaire (SFFQ)	Caffeine was 137 mg per cup of coffee, 47 mg per cup of tea, 46 mg per can or bottle of cola beverage, and 7 mg per serving of chocolate candy.	Self-report and medical records
Ascherio et al. [22]	USA	Nurses’ Health Study (1976/1998)	121,700 women	Semiquantitative food-frequency questionnaire (SFFQ)	Caffeine was 137 mg per cup of coffee, 47 mg per cup of tea, 46 mg per can or bottle of cola beverage, and 7 mg per serving of chocolate candy.	Medical records
Grandinetti et al. [21]	USA	Honolulu Heart Program-Japanese and Okinawan ancestry (1965/1991)	8006 men	Questionnaires	NA	Medical records
Hu et al. [23]	FIN	Four independent cross-sectional population surveys were carried out in five geographic areas of Finland in 1982, 1987, 1992, and 1997 (1982/2002)	29,335	Self-administered questionnaire	Cups of coffee	National Social Insurance Institution’s Register
Liu et al. [18]	USA	NIH-AARP Diet and Health Study (1995/2010)	566,401	Diet History Questionnaire	Nutrient calculation: 1994–1996 US Department of Agriculture’s Continuing Survey of Food Intakes by Individuals.	Interview and copy of medical records
Palacios et al. [25]	USA	CPS II–Nutrition cohort (1992/2007)	184,190	Food Frequency Questionnaire	137 and 47 mg per cup of coffee and tea, respectively, 46 mg per can or bottle of cola; and 7 mg per serving of chocolate.	Interview and copy of medical records
Sääksjärvi et al. [15]	FIN	Finnish Mobile Clinic Health Examination Survey (1973/1994)	7246	Self-administered, health questionnaire	Cups of coffee	National Social Insurance Institution’s Register
Tan et al. [24]	SG	Singapore Chinese Health Study (1993/2005)	63,257	A validated, semiquantitative food frequency section questionnaire	Singapore Food Composition Table, a food-nutrient database that lists the levels of 96 Nutritive/nonnutritive components (including caffeine) per 100 g of cooked food and beverages	Interview and linkage database to medical record
Wirdefeldt et al. [16]	SE	Swedish Twin Registry (1961 and 1973/without clear mentioning)	52,149	Questionnaires	Did not provide the formula	Inpatient Discharge Register and Cause of Death Register

**Table 2:** List of the included studies on the progression of Parkinson’s disease (PD).

Study Name	Country	Number of PD	Stage of PD	Assessment Caffeine Consumption	Amount of Caffeine Consumption	Mean Follow-Up Period of Time	Outcome as the Progression of PD
Kandinov et al. [26]	IL	278	Onset of PD motor symptoms	Interview	The number of cups of coffee per day	10.3 years	Time from onset to Hoehn and Yahr stage 3
Moccia et al. [29]	IL	79	de novo, drug naïve	Caffeine Consumption Questionnaire	i.e., Espresso 1oz = 50 mg caffeine	4 years	Starting L-dopa treatment
Scott et al. [28]	GB	183	Newly diagnosed	Verbal interview about the average level of exposure before baseline	Cups of tea: 47 mg caffeine Cup of coffee: 62 mg caffeine	59 months	1.Motor fluctuation 2.Dyskinesia
Wills et al. [27]	US	228	Early PD	questionnaire assessing both current (“in the past week”) and prior (“on average over the past 5 years”) caffeine intake	Coffee (85 mg caffeine/5 oz) Tea (36 mg caffeine/5 oz) Soda (45 mg caffeine/12 oz)	5.5 years	Dyskinesia
